# Chromosome spreading of associated transposable elements and ribosomal DNA in the fish Erythrinus erythrinus. Implications for genome change and karyoevolution in fish

**DOI:** 10.1186/1471-2148-10-271

**Published:** 2010-09-06

**Authors:** Marcelo B Cioffi, Cesar Martins, Luiz AC Bertollo

**Affiliations:** 1 Universidade Federal de São Carlos, Departamento de Genética e Evolução, São Carlos, SP, Brazil; 2 UNESP- Universidade Estadual Paulista, Instituto de Biociências, Departamento de Morfologia, Botucatu, SP, Brazil

## Abstract

**Background:**

The fish, *Erythrinus erythrinus*, shows an interpopulation diversity, with four karyomorphs differing by chromosomal number, chromosomal morphology and heteromorphic sex chromosomes. Karyomorph A has a diploid number of 2n = 54 and does not have differentiated sex chromosomes. Karyomorph D has 2n = 52 chromosomes in females and 2n = 51 in males, and it is most likely derived from karyomorph A by the differentiation of a multiple X_1_X_2_Y sex chromosome system. In this study, we analyzed karyomorphs A and D by means of cytogenetic approaches to evaluate their evolutionary relationship.

**Results:**

Conspicuous differences in the distribution of the 5S rDNA and *Rex3 *non-LTR retrotransposon were found between the two karyomorphs, while no changes in the heterochromatin and 18S rDNA patterns were found between them. *Rex3 *was interstitially dispersed in most chromosomes. It had a compartmentalized distribution in the centromeric regions of only two acrocentric chromosomes in karyomorph A. In comparison, in karyomorph D, *Rex3 *was found in 22 acrocentric chromosomes in females and 21 in males. All 5S rDNA sites co-localized with *Rex3*, suggesting that these are associated in the genome. In addition, the origin of the large metacentric Y chromosome in karyomorph D by centric fusion was highlighted by the presence of internal telomeric sites and 5S rDNA/*Rex3 *sites on this chromosome.

**Conclusion:**

We demonstrated that some repetitive DNAs (5S rDNA, *Rex3 *retroelement and (TTAGGG)_n _telomeric repeats) were crucial for the evolutionary divergence inside *E. erythrinus*. These elements were strongly associated with the karyomorphic evolution of this species. Our results indicate that chromosomal rearrangements and genomic modifications were significant events during the course of evolution of this fish. We detected centric fusions that were associated with the differentiation of the multiple sex chromosomes in karyomorph D, as well as a surprising increase of associated 5S rDNA/*Rex3 *loci, in contrast to karyomorph A. In this sense, *E. erythrinus *emerges as an excellent model system for better understanding the evolutionary mechanisms underlying the huge genome diversity in fish. This organism can also contribute to understanding vertebrate genome evolution as a whole.

## Background

Repetitive DNA sequences include tandemly-arrayed satellites, as well as minisatellites, microsatellites and dispersed repeats such as transposable elements (TEs) [1]. Satellite DNAs are organized as long arrays of head-to-tail linked repeats. TEs are DNA segments capable of integrating into new locations in the genome, and they also mobilize non-autonomous sequences [2,3]. TEs and satellite DNAs are some of the most important components of the genome that contribute to genetic variations within and between species [4]. The possible functions of these repetitive DNAs have been the focus of several studies, and there are indications that they could play important roles at both the chromosomal and nuclear levels [5-8].

Fish genomes contain all known types of transposable elements: classical DNA transposons, miniature inverted-repeat transposable elements and retroelements, which include long terminal repeat (LTR) retrotransposons and non-LTR retrotransposons [9]. While DNA transposons move directly as DNA molecules from one genomic site to another, retroelements transpose via an RNA intermediate. Among retrotransposable elements, *Rex *is comprised of various families of transposable elements that are abundant in teleosts. *Rex3*, the first reverse transcriptase (RT)-encoding retrotransposon isolated from the melanoma fish model, *Xiphophorus*, is a non-LTR element related to the RTE family that shows wide distribution and different patterns of organization in the genomes of several fish species [10,11].

The molecular organization and cytogenetic locations of repetitive DNAs, including rDNA repeats [12-18], satellite DNAs [19,20], telomeric sequences [18,21,22] and several classes of TEs [2,3,23,24], have been analyzed in a large number of fish species. These studies have demonstrated the enormous potential that the investigation of repetitive DNAs offers toward extending our knowledge of karyotype differentiation and sex chromosome evolution in fish [16,18,25-29]. These genomic components are able to change the molecular composition of sex chromosomes and reduce the rate of recombination between them, which are crucial steps in the differentiation of sex chromosomes [30-33].

*Erythrinus *is a cytogenetically poorly studied genus inside the Erythrinidae family. Until now, classical cytogenetic analyses have only been conducted with the species, *E. erythrinus*. These have shown a karyotypic diversity among populations, with four currently identified karyomorphs (A to D) [34]. Karyomorph A is comprised of populations with 2n = 54 chromosomes, which have very similar karyotypic structures (6m + 2st + 46a) and an absence of differentiated sex chromosomes. Karyomorphs B, C and D share an X_1_X_1_X_2_X_2_/X_1_X_2_Y sex chromosome system, but they differ in their diploid number and chromosomal morphology. It has been proposed that a centric fusion between two non-homologous acrocentric chromosomes may have created the specific Y chromosome and, consequently, the unpaired X_1 _and X_2 _chromosomes in the male karyotypes. Karyomorph B has 2n = 54 (6m + 2st + 46a) chromosomes in females and 2n = 53 (7m + 2st + 44a) in males. Both karyomorphs C and D show 2n = 52/51 chromosomes but differ in their karyotypic formula, i.e., 6m + 2sm + 6st + 38a in females and 7m + 2sm + 6st + 36a in males of karyomorph C and 4m + 2sm + 2st + 44a in females and 5m + 2sm + 2st + 42a in males of karyomorph D. The distinct chromosomal features found among isolated populations suggest the occurrence of several unnamed new species within this fish group [34].

In this report, new samples from allopatric populations of karyomorphs A and D were analyzed using new methodological approaches and molecular cytogenetic analyses to find useful new characteristics for comparative genomics at the chromosomal level and to provide insights into the karyoevolutionary pathways in this fish group. The results show that chromosomal rearrangements and genomic modifications were significant events during the course of evolution of this fish. Centric fusions were found to be clearly associated with the differentiation of the multiple sex chromosomes in karyomorph D. In addition, a surprising increase in the number of associated 5S rDNA/*Rex3 *loci was found in karyomorph D, in contrast to karyomorph A.

## Results

### Karyotyping and C-banding

**Figure 1:**
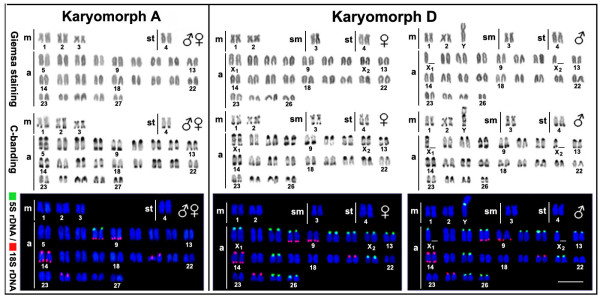
**Karyotypes of males and females of *Erythrinus erythrinus (*karyomorphs A and D) under different cytogenetic analyses**. The karyotypes, arranged by sequentially Giemsa-stained and C-banded chromosomes, were probed with 5S rDNA and 18S rDNA after a double-FISH analysis. Note the significant increase of 5S rDNA sites in karyomorph D. m, metacentric chromosomes; sm, submetacentric chromosomes; st, subtelocentric chromosomes; a, acrocentric chromosomes. Bar = 5 μm.

The two populations showed evidence of the general karyotypic structures of the *Erythrinus *species, with few biarmed chromosomes and a large number of acrocentric ones (Fig. 1). The sample from Penápolis-SP showed 2n = 54 chromosomes (6m + 2st + 46a) and lacked morphologically differentiated sex chromosomes, which is characteristic of karyomorph A. The samples from Natal-RN showed 2n = 52 chromosomes (4m + 2sm + 2st + 42a) in females and 2n = 51 chromosomes (5m + 2sm + 2st + 42a) in males, with a multiple X_1_X_1_X_2_X_2_/X_1_X_2_Y sex chromosome system, which is characteristic of karyomorph D (Fig. 1). Conspicuous C-positive bands were observed in the centromeric/pericentromeric region of several chromosomes, as well as in the telomeric region of some pairs, in both karyomorphs. A small but significant heterochromatic block was found in the interstitial region of the long arms of the Y and X_1 _chromosomes of karyomorph D (Fig. 1).

### Nucleotide sequences

Nucleotide sequences were determined for the *Rex3 *clones to confirm that the PCR-isolated DNA fragments corresponded to copies of the retrotransposable element, *Rex3*. One of these sequences was deposited in GenBank under the accession number, GU989321. NCBI BlastN searches identified a similarity between the *E. erythrinus Rex3 *sequence and sequences found in fish from other distinct orders, such as Anguilliformes, Perciformes, Beloniformes, Cyprinodontiformes and Tetraodontiformes.

### *Cytogenetic mapping *of 18S and 5S rDNAs, Rex3 and (TTAGGG)_n _telomeric repeats

Double-FISH with 5S and 18S rDNAs showed a similar distribution pattern for the 18S rDNA sites in both the A and D karyomorphs. Five acrocentric pairs with telomeric sites on the long or short arms were found. In addition, bitelomeric sites were found on pair no. 14. In contrast, a large difference was seen in 5S rDNA distribution. Karyomorph A showed only two 5S rDNA sites in the centromeric region of acrocentric pair no. 8, which also bears a telomeric 18S rDNA site on its long arm. Although karyomorph D shared the syntenic condition seen in karyomorph A, it showed a surprising increase in the number of 5S rDNA sites, with 22 in females and 21 in males. These sites were all found in the centromeric region of acrocentric chromosomes except for a site in the metacentric Y chromosome in males (Fig. 1).

**Figure 2:**
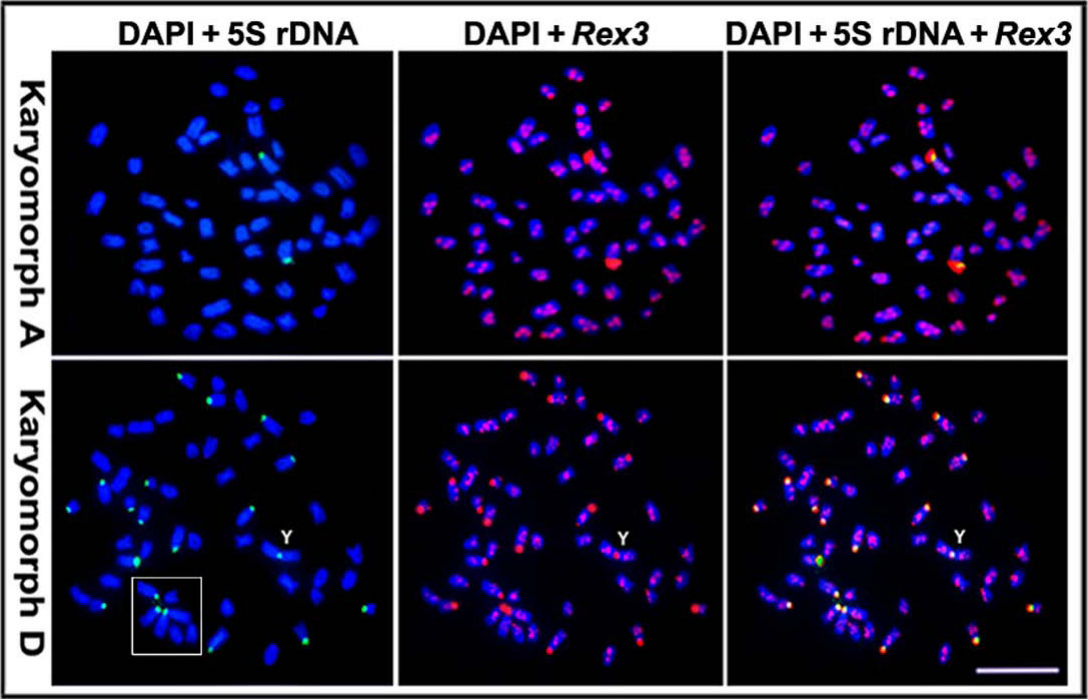
**Metaphase plates of karyomorphs A and D of *Erythrinus erythrinus *showing the locations of the 5S rDNA and the *Rex3 *retroelement on the chromosomes using double-FISH analysis**. Note the dispersed interstitial pattern of *Rex3 *in both karyomorphs and its co-localization with the 5S rDNA on the centromeric region of the chromosomes. The box indicates the clear aggregation of some acrocentric chromosomes, which was seen in almost all chromosome preparations. The Y chromosome is indicated. Bar = 5 μm.

Double-FISH with 5S rDNA and *Rex3 *probes showed that *Rex3 *has an interstitial and dispersed distribution pattern along most chromosomes in both karyomorphs. In addition, *Rex3 *clusters were predominantly located in the centromeric regions and co-localized with heterochromatic blocks in both karyomorphs, which matches the 5S rDNA distribution pattern (Fig. 2).

**Figure 3:**
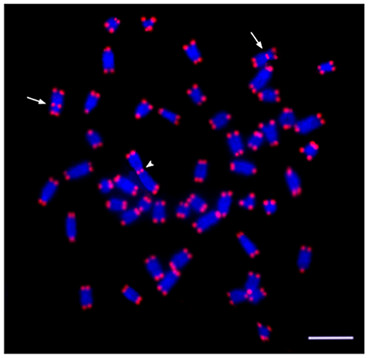
**Male metaphase plate of *Erythrinus erythrinus *karyomorph D showing the location of the telomeric hybridization signals on both telomeres of all chromosomes**. In addition, ITS are found in the centromeric region of the only submetacentric pair in the karyotype (arrows) and on the large metacentric Y chromosome (arrowhead). Bar = 5 μm.

Mapping of the (TTAGGG)_n _telomeric repeats in males of karyomorph D showed the typically expected telomeric signals on both telomeres of all chromosomes. Interstitial telomeric sites (ITS) were located in the centromeric regions of the only submetacentric pair and on the largest metacentric Y chromosome (Fig. 3).

## Discussion

Among Characiformes fish, which include the Erythrinidae family, the most frequent chromosomal number is 2n = 54, and this number may represent the basal diploid number of this order [35]. In this context, karyomorph A of *E. erythrinus*, which has a diploid number of 2n = 54, may have the most primitive karyotype found in the *Erythrinus *genus. This finding also takes into account the fact that differentiated sex chromosomes are absent in this karyomorph. Similarly, karyomorph D, which is most likely derived from karyomorph A, shows a smaller diploid number due to chromosomal rearrangements and a well-differentiated multiple X_1_X_1_X_2_X_2_/X_1_X_2_Y sex chromosome system. However, despite differences in diploid numbers and the occurrence of differentiated sex chromosomes, karyomorphs A and D share a relatively similar karyotypic structure formed by several acrocentric and a few biarmed chromosomes with similarly distributed C-bands and 18S rDNA sites. However, the repetitive 5S rDNA and *Rex3 *sequences have quite distinct distributions in the two karyomorphs.

**Figure 4:**
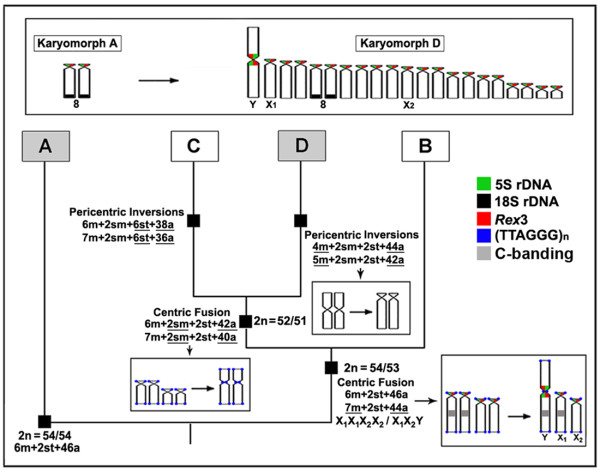
**Overview of the evolutionary karyotypic pathways proposed for karyomorphs A-D of *Erythrinus erythrinus *on the basis of their karyotypic features and FISH mapping results for karyomorphs A and D**. The main chromosomal changes from the probable basal karyotype (2n = 54; 6m + 2st + 46a) are underlined. The upward schematic figure illustrates the chromosomal distribution of the 18S rDNA and 5S rDNA/*Rex3 *sequences in karyomorph A and the expanded distribution of the latter sites in karyomorph D, including the Y, X_1 _and the probable X_2 _chromosome. The different colors represent the probes used for FISH.

A reduction in chromosome numbers often results from centric fusion rearrangements from acrocentric chromosomes. Based only on classical cytogenetic data, it was previously proposed that an initial centric fusion gave rise to the X_1_X_1_X_2_X_2_/X_1_X_2_Y sex system found in karyomorphs B, C and D of *E. erythrinus*. Also, it was proposed that the differentiation of karyomorph D resulted from another centric fusion between two nonhomologous acrocentric chromosomes, which was the origin of the only submetacentric pair found in the karyotype. In addition, pericentric inversions completed this karyotypic differentiation by decreasing the number of metacentric chromosomes and increasing the number of acrocentric chromosomes [34]. Indeed, this karyomorphic differentiation in *E. erythrinus *has now been corroborated by our FISH mapping of repetitive DNA sequences (Fig. 4).

The chromosomal location of 5S rDNA, *Rex3 *and telomeric repeats clearly corroborates the centric fusions that occurred during the karyotypic differentiation of karyomorph D. The mapping of ITS in the centromeric region of the submetacentric pair highlights the centric fusion that was involved in the origin of this chromosome pair, which is not found in karyomorph A. Similarly, our results support the proposed origin for the largest metacentric Y chromosome from another centric fusion. As expected, (TTAGGG)_n _repeats were also found in the centromeric region of this chromosome (Fig. 3). ITS have been found in the centromeric region of a large number of vertebrate species, suggesting that chromosomal rearrangements can occur without the loss of these telomeric sequences [36]. The general hypothesis that ITS may be remnants of chromosome rearrangements that occurred during genome evolution is supported by several investigations [37].

The location of 5SrDNA/*Rex3 *sequences at the centromeric position of the Y chromosome is of particular relevance. These sequences were found in the centromeric region of several acrocentric chromosomes, including the ones proposed as X_1 _(no. 5) and X_2 _(no. 12) in the karyotype. The mapping of 5S rDNA/*Rex3 *sites in the centromeric region of the Y chromosome suggests that this chromosome was created from a centric fusion of acrocentric pairs (nos. 5 and 12), which gave rise to the unpaired X_1 _and X_2 _chromosomes in the male karyotype (Figs. 1 and 2). Although the identification of the X_2 _chromosome remains unclear, the X_1 _chromosome appears to be the first acrocentric pair (no. 5) in this karyotype. This result is supported by the C-banding pattern in which a faint but informative C-positive band occurs interstitially in corresponding regions of the X_1 _chromosome and the long arm of the Y chromosome. It is probable that the same centric fusion also gave rise to the X_1_X_2_Y sex system in karyomorphs B and C, since it appears to have originated before the divergence of these three karyomorphs [34] (Fig. 4).

The most remarkable difference between karyomorphs A and D was the distribution of 5S rDNA/*Rex3 *sites over the chromosomes. While only a single chromosome pair was found to bear these sites in karyomorph A, a surprisingly large number of these sites were found in karyomorph D, with 22 sites in females and 21 in males. (Figs 2 and 4). *Rex3*, a non-LTR retrotransposon first isolated from the platyfish, *Xiphophorus maculatus*, shows a wide distribution among teleost fishes, where it appears to be associated with the evolution of different lineages [10,11]. Although *Rex3 *shows a preferential localization in the centromeric region of chromosomes in some fishes [23,24,38-40], it is widely scattered over all chromosomes in several Antarctic ice-fish species, with intense hybridization signals in some specific chromosomal regions [3]. Since a large majority of fish species studied until now harbor a low number of 5S rDNA sites, with a few exceptions [41,42], the high number of 5S rDNA sites found in karyomorph D was an intriguing feature. According to the most probable hypothesis that karyomorph D represents a derivative form compared to karyomorph A, our results clearly show a huge dispersal of 5S rDNA/*Rex3 *elements throughout the centromeric regions of the acrocentric chromosomes. We hypothesize that *Rex3 *may have inserted into 5S rDNA sequences and that the 5S rDNA-*Rex3 *complex moved and dispersed in the karyotype, although this hypothesis deserves further investigation and a molecular characterization. The clear association among the centromeric regions of the acrocentric chromosomes appears to be a favorable condition for this spreading (see details in Fig. 2).

Previous reports have suggested that the rDNA locus can serve as an ideal niche for the long-term survival of TEs [43], as seen in several organisms [44-46]. *In situ *hybridization revealed permanent clustering of different TEs in the NOR regions, as well as near or within clusters of 5S rDNA [45,47]. Hybridization on extended DNA fibers identified insertions of TEs inside the rDNA region, the overlap of rDNA and TE-enriched regions, and small fragments of rDNA inside TE-enriched regions. The presence of TEs in or around rDNA sites increases the possibility for recombination, which appears to be a common event in plant karyotype evolution [48]. In flowering plants, the distribution of 5S rDNA genes is highly variable and may be partially explained by the activity of small non-autonomous retrotransposons named Cassandra [49]. It is currently believed that TEs tend to accumulate in heterochromatic regions because there are fewer genes and a weaker selection in the heterochromatin than in the euchromatin [50]. Recent studies have proposed that the activity of TEs is one possible source for rDNA movement [43,48]. Studies have also documented the ability of some classes of transposons to capture entire genes and move them to different parts of the genome [51,52].

Thus, considering the correlation between karyotype rearrangement and retrotransposon activity [3], and that rapid chromosomal evolution in some vertebrate lineages may be driven by the activity of repetitive sequences [53], we propose two probable alternatives during the karyotypic diversification of *E. erythrinus*, *i.e*., (i) the chromosomes of this species have undergone rearrangements during an evolutionary process mediated by retrotransposon activity or (ii) rearrangement events, including posterior mobilization of TEs, promoted the karyotypic differentiation among populations. However, it is difficult to state if a change in TE content or activity is the cause or the consequence of a speciation process because the true role of transposable elements in speciation is still a subject of large debate [6].

The frequent switching between different sex determination systems and the rapid evolution of sex chromosomes in fishes may also be linked to the formation of new species [54]. Recent comparative studies have revealed that teleost genomes have experienced a higher rate of gene-linkage disruption and chromosomal rearrangements compared to mammals, which may be linked to the apparent plasticity of their genomes [55,56]. Studies in fish models would therefore help in better understanding the molecular and evolutionary mechanisms underlying the huge genome diversity in this group, and these studies would also contribute to our understanding of vertebrate genome evolution.

## Conclusion

Our in situ investigation of repetitive DNA sequences resulted in useful new characteristics for comparative genomics at the chromosomal level and provided insights into the karyoevolutionary pathways in *E. erythrinus *fish. Chromosomal rearrangements and genomic modifications were significant events during the course of karyoevolution of this fish. The spreading of associated transposable elements and ribosomal DNA in the genome and the differentiation of a multiple sex chromosome system was strongly associated with the evolution of karyomorph D. Considering the facts that fish occupy the basal position in the phylogeny of vertebrates, that they have a diversity of sex determining mechanisms and that many fish species lack heteromorphic sex chromosomes, *E. erythrinus *emerges as an excellent model system for better understanding the evolutionary mechanisms underlying the huge genome diversity found among vertebrates.

## Methods

### Specimens, mitotic chromosome preparation, chromosome staining and karyotyping

In this study, we analyzed new samples from populations of karyomorphs A and D of the fish, *E. erythrinus*. We studied a total of 28 specimens (16 males and 12 females). Overall, 13 specimens (8 males and 5 females) of karyomorph A were obtained from Penápolis - São Paulo State, and 15 specimens (8 males and 7 females) of karyomorph D were obtained from Natal - Rio Grande do Norte State. These samples belong to distinct Brazilian hydrographic basins, which are isolated by thousands of kilometers.

Mitotic chromosomes were obtained from cell suspensions of the anterior kidney using the conventional air-drying method [57]. The experiments followed ethical conducts, and anesthesia was used prior to sacrificing the animals. The process was approved by the FAPESP committee under no. 2009/14881-3. Chromosomes were sequentially Giemsa-stained and C-banded using barium hydroxide to detect the C-positive heterochromatin [58]. Approximately 30 metaphase spreads were analyzed per specimen to determine the diploid chromosome number and karyotype structure. Images were captured by the CoolSNAP system software, Image Pro Plus, 4.1 (Media Cybernetics, Silver Spring, MD, USA), coupled to an Olympus BX50 microscope (Olympus Corporation, Ishikawa, Japan). The chromosomes were classified as metacentric (m), submetacentric (sm), subtelocentric (st) or acrocentric (a) according to the arm ratios [59].

### Chromosome probes

Two tandemly-arrayed DNA sequences isolated from the genome of another Erythrinidae species, *Hoplias malabaricus*, were used. The first probe contained a 5S rDNA repeat copy and included 120 base pairs (bp) of the 5S rRNA transcribing gene and 200 bp of the non-transcribed spacer (NTS) [12]. The second probe corresponded to a 1,400-bp segment of the 18S rRNA gene obtained via PCR from nuclear DNA [16]. The 5S and 18S rDNA probes were cloned into plasmid vectors and propagated in DH5α *E. coli *competent cells (Invitrogen, San Diego, CA, USA).

The retrotransposable element, *Rex3*, was obtained by PCR directly from the genome of *E. erythrinus *using the primers *Rex3*f (5'-CGG TGA YAA AGG GCA GCC CTG) and *Rex3*r (5'-TGG CAG ACN GGG GTG GTG GT- 3'), as previously described [2,3]. The obtained nucleotide segment of the *Rex3 *transposon corresponds to the encoding domains 1, 2, 2A, A and B of the RT gene [2]. A PCR-generated amplicon (~500 bp) was isolated from a gel, purified with the Sephaglas Band Prep Kit (Pharmacia Biotech, Orsay, France) and ligated into the pGEM-T plasmid (Promega, Heidelberg, Germany). This plasmid was used to transform DH5α *E. coli *competent cells (Invitrogen, San Diego, CA, USA). The positive clones were sequenced on an ABI Prism 377 DNA sequencer (Perkin Elmer, Branchburg, NJ, USA) with the ABI Prism BigDye Terminator Cycle Sequencing Ready Reaction Kit (Perkin Elmer, Branchburg, NJ, USA). The nucleotide sequence was subjected to Blastn [60] searches at the National Center for Biotechnology Information (NCBI) website http://www.ncbi.nlm.nih.gov/blast for the identification of any similarity of the isolated sequences to any known sequences from the nucleotide collection (nt/nr), whole-genome shotgun reads (WGS), genomic survey sequences (GSS) and high-throughput genomic sequences (HTGS) in GenBank.

The 5S rDNA probe was labeled with biotin-14-dATP by nick translation according to the manufacturer's recommendations (BioNick™Labeling System; Invitrogen, San Diego, CA, USA). The 18S rDNA and *Rex3 *probes were labeled by nick translation with DIG-11-dUTP according to the manufacturer's instructions (Roche, Mannheim, Germany).

A probe from the telomeric DNA sequence (TTAGGG)_n _was generated by PCR (PCR DIG-Probe Synthesis Kit, Roche) in the absence of a template using (TTAGGG)_5 _and (CCCTAA)_5 _as primers [37].

Fluorescent *in situ *hybridization (FISH) was performed under high stringency conditions on mitotic chromosome spreads [61]. The metaphase chromosome slides were incubated with RNAse (40 μg/ml) for 1.5 h at 37°C. After denaturation of chromosomal DNA in 70% formamide/2× SSC at 70°C, spreads were incubated in 2× SSC for 4 min at 70°C. The hybridization mixture (2.5 ng/μl probes, 2 μg/μl salmon sperm DNA, 50% deionized formamide, 10% dextran sulphate) was dropped on the slides, and the hybridization was performed overnight at 37°C in a moist chamber containing 2× SSC. Two post-hybridization washes were carried out on a shaker (150 rpm) at 37°C. The first wash was in 2× SSC, 50% formamide for 15 min, followed by a second wash in 2× SSC for 15 min. A final wash was performed at room temperature in 4× SSC for 15 min. Avidin-FITC (Sigma, St. Louis, MO, USA) was used for signal detection of the 5S rDNA probe and anti-digoxigenin-rhodamine (Roche, Mannheim, Germany) for 18S rDNA, *Rex3 *and (TTAGGG)_n _probes. One-color FISH was performed to detect (TTAGGG)_n _repeats, while 5S/18S rDNA and 5S rDNA/*Rex3 *were detected by double-FISH. The chromosomes were counterstained with DAPI (1.2 μg/ml), mounted in Antifade solution (Vector, Burlingame, CA, USA) and analyzed in an epifluorescence microscope Olympus BX50 (Olympus Corporation, Ishikawa, Japan)

## Abbreviations

2n: diploid number; DAPI: 4'-6-Diamidino-2-phenylindole; FITC: fluorescein isothiocyanate; FISH: fluorescent in-situ hybridization; ITS: interstitial telomeric sites; LTR: long terminal repeats; NCBI: National Center for Biotechnology Information; NOR: nucleolar organizer region; NTS: non-transcribed spacer; PCR: polymerase chain reaction; RT: reverse transcriptase; rDNA: ribosomal DNA; SSC: sodium chloride-sodium citrate buffer; TEs: transposable elements.

## Authors' contributions

MBC performed the experiments and drafted the manuscript. CM helped in analysis and drafted the manuscript. LACB designed and coordinated the study, and drafted and revised the manuscript. All authors read and approved the final manuscript.
